# Modeling and Solution of Large Amplitude Vibration Problem of Construction Elements Made of Nanocomposites Using Shear Deformation Theory

**DOI:** 10.3390/ma14143843

**Published:** 2021-07-09

**Authors:** Ali Deniz, Nicholas Fantuzzi, Abdullah Heydaroglu Sofiyev, Nuri Kuruoglu

**Affiliations:** 1 Department of Mathematics, Faculty of Arts and Sciences, Usak University, 64200 Usak, Turkey; 2 Department of Civil, Chemical, Environmental, and Materials Engineering, University of Bologna, Viale Risorgimento 2, 40136 Bologna, Italy; nicholas.fantuzzi@unibo.it; 3 Department of Civil Engineering, Engineering Faculty, Suleyman Demirel University, 32260 Isparta, Turkey; abdullahavey@sdu.edu.tr; 4 Department of Civil Engineering, Faculty of Engineering and Architecture, Istanbul Gelisim University, 34310 Istanbul, Turkey; nkuruoglu@gelisim.edu.tr

**Keywords:** CNT, nanocomposite constructions, shear deformation theory, large amplitude frequency

## Abstract

The main purpose of the study is to investigate the vibration behaviors of carbon nanotube (CNT) patterned double-curved construction elements using the shear deformation theory (SDT). After the visual and mathematical models of CNT patterned double-curved construction elements are created, the large amplitude stress–strain relationships and basic dynamic equations are derived using the first order shear deformation theory (FSDT). Then, using the Galerkin method, the problem is reduced to the nonlinear vibration of nanocomposite continuous systems with quadratic and cubic nonlinearities. Applying the Grigolyuk method to the obtained nonlinear differential equation, large-amplitude frequency-amplitude dependence is obtained. The expressions for nonlinear frequencies of homogenous and inhomogeneous nanocomposite construction members such as plates, panels, spherical and hyperbolic-paraboloid (hypar) shells in the framework of FSDT are found in special cases. The accuracy of the results of the current study has been confirmed by comparing them with the reliable results reported in the literature. Original analyses are carried out to examine the effects of nonlinearity, CNT patterns and volume fraction changes on frequencies in the framework of shear deformation and classical shell theories.

## 1. Introduction

Theoretical studies on the analysis of dynamic behavior of thin and moderately thick construction elements are based on several provisions of geometric and physical nature. One of the most important geometric features of construction elements is the mid-surface, which is taken as a design model. In contemporary technology, mainly such shells are used, the middle surface of which is determined by continuous and adequately differentiable functions. One of these types of construction elements is double-curved shells. The fact that double-curved shell-type constructions have a wide area of use under dynamic loads in various industries as the main construction element has made it advisable to investigate their large amplitude behavior in the framework of developed theories and to find new solution methods. Although the classical shell theory (CST) gives reasonable results for thin homogeneous isotropic construction elements, it turns out that relatively thick composite shells do not give sufficiently realistic results in vibration problems. This factor prompted researchers to use shear deformation theories instead of classical shell theory for relatively thick construction elements in nonlinear vibration problems. In addition to the assumptions in the classical shell theory, various shear deformation theories (SDTs) have been developed considering the effect of transverse shear deformations [1,2,3]. In the framework of SDTs, derivation and solution of nonlinear basic differential equations of shell-type construction elements becomes significantly complicated. For these reasons, the number of studies on the solution of linear and nonlinear vibration problems of construction elements consisting of traditional and new generation composites is greater in the framework of CST.

Significant advances in nanoscale science and technology in the last two decades have led to the production of various nanoscale materials and have enabled the discovery of their superior physical, optical, electrical and mechanical properties. CNTs, one of such nanoscale materials, have attracted great interest in application in various fields of modern technology due to their outstanding properties [4,5,6,7].

One of the most important engineering applications of carbon nanotubes is that they can be used as reinforcing elements in polymer matrices. The design and modeling of CNT patterned composites, physical interactions at the CNT-polymer interface and the determination of macroscopic elastic properties of CNT patterned polymers have been studied by many researchers [8,9,10,11,12]. CNT patterned polymer composites have been widely used in the mechanical engineering, automotive and marine industries over the past few decades due to their low weight and superior mechanical properties. As aerospace engineering is considered one of the leading disciplines of the future, nanocomposites represent a new generation of functionally graded materials that could change the traditional structure of this favorite industry. With the shuttle decommissioning and the wider use of composite materials in modern and future commercial aircraft such as the Boeing 787 and Airbus A380, the potential applications and benefits of functionally graded nanocomposites in the aeronautical and aerospace industry are today indisputable [13,14,15].

The first attempt at the formulation, mathematical modeling and solution of nonlinear problems of CNT patterned composite construction elements was made in 2009, and this research served as a guide for solving various stability and vibration problems in the following periods (see Shen [16]). Following this study, studies on the solution of linear and nonlinear vibration problems of CNT patterned construction elements continue until the present day. Pouresmaeeli and Fazelzadeh [17] presented the linear frequency analysis of doubly curved functionally graded carbon nanotube-reinforced composite panels based on the FSDT. Wang et al. [18,19] studied the linear vibration of CNT patterned composite double-curved panels and shells of revolution using FSDT. Braun et al. [20] presented bulk modulus and natural frequency of fullerene and nanotube carbon structures using the Galerkin method and Abaqus software. Ansari et al. [21] investigated flexural and free vibration analysis of CNT-reinforced functionally graded plate. Tran et al. [22] studied free vibration analysis of smart laminated functionally graded CNT reinforced composite plates via new four-variable refined plate theory. Qin et al. [23] presented a general approach for linear free vibration analysis of CNT patterned cylindrical shells with arbitrary boundary conditions by using Chebyshev polynomials. Sofiyev and coauthors studied linear free and forced vibration and stability problems of CNT patterned cylindrical and conical shells based on the classical and shear deformation shell theories in different media [24,25,26]. Azarafza et al. [27] investigated the linear free vibration of CNT- patterned grid-stiffened composite cylindrical shell within FSDT and using the ABAQUS CAE software. Cornacchiaet al. [28] presented an analytical solution of linear vibrations and buckling of cross- and angle-ply nano plates using strain gradient theory. Vinyas et al. [29] reported the linear vibration analysis of CNT patterned magneto-electro-elastic plates with different electromagnetic conditions using higher order finite element methods.

In the abovementioned studies, the vibration behavior of CNT patterned constructions is formulated in small displacements, and linear frequency values are obtained in different environments and using various approaches. Large displacements or geometrical nonlinearity in constructions composed of CNT patterned polymers create considerable qualitative and quantitative changes in their dynamic behaviors, as well as complicating modeling, solutions and analysis. Although these reasons limited the number of publications on the nonlinear problems of CNT patterned constructions in the early years, research on this subject has gained momentum in recent years. Shen and Xiang [30] proposed a solution to nonlinear vibration of composite cylindrical shells patterned with nanotubes in thermal environment. Nguyen et al. [31] presented nonlinear vibrations and dynamic response of composite truncated conical shells with CNT patterns in various media using a two-component deflection function. Zghal et al. [32] presented large deflection response-based geometrical nonlinearity of CNT pattern nanocomposite structures using a finite element method. Dat et al. [33] used an analytical approach to solve the nonlinear magnetoelastic vibration of an intelligent sandwich plate on an elastic foundation. Zhang et al. [34] analyzed the geometrically nonlinear behavior of CNT patterned composite plates with piezoelectric layers. Huang et al. [35] presented geometric nonlinear analysis of auxetic hybrid laminated beams containing CNT reinforced composite materials. Avey and Yusufoglu [36], within the framework of classical shell theory, studied large-amplitude vibrations of double-curved shallow shells based on carbon nanotubes. Yusufoglu and Avey [37] presented nonlinear dynamic behavior of hyperbolic paraboloidal shells reinforced by carbon nanotubes with various distributions. Chakraborty and Day [38] investigated a nonlinear stability analysis of CNT-patterned composite cylindrical shells subjected to the thermomechanical load. Yadav et al. [39] investigated a semi-analytical solution of nonlinear vibrations of CNT patterned circular cylindrical shells using the harmonic balance method. Liew et al. [40] examined and evaluated in detail the latest developments of functionally graded CNT-reinforced composites and structures.

In the abovementioned studies, the nonlinear behavior of CNT patterned construction elements is generally discussed using numerical methods, while analytical solutions are presented within the framework of classical shell theory. The sensitive and complex nature of nanocomposite double-curved construction elements further increases the difficulty of mathematical operations in the formulation, modeling and solution of their nonlinear vibration problems in the framework of SDTs. These factors prevented the nonlinear free vibration problem of CNT patterned composite construction members from being adequately investigated analytically. In this research, we aim to solve the problem in an analytical way.

## 2. Formulation of Problem

Mathematical Modelling of Constructions with CNT Patterns.

**Figure 1:**
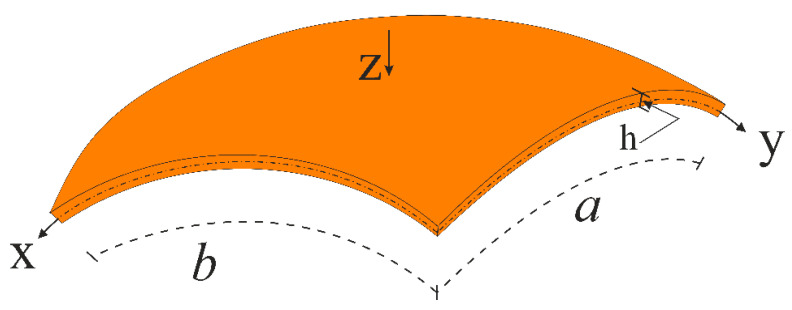
Double-curved shallow shell and coordinate system.

Figure 1 shows a double-curved CNT patterned shell with the xyz coordinate system located on the mid-surface, the origin of which is in the left corner. The z coordinate is normal to the xy surface and is directed towards the inside of the shell. The lengths of the shell in the x and y directions are indicated as a and b, respectively, and the thickness as h. In the chosen coordinate system, shallow shells are defined as the three-dimensional region,Λ, as follows:

(1) Λ={x,y,z:(x,y,z)∈[0,a]×[0,b]×[−h/2,h/2]}

**Figure 2:**
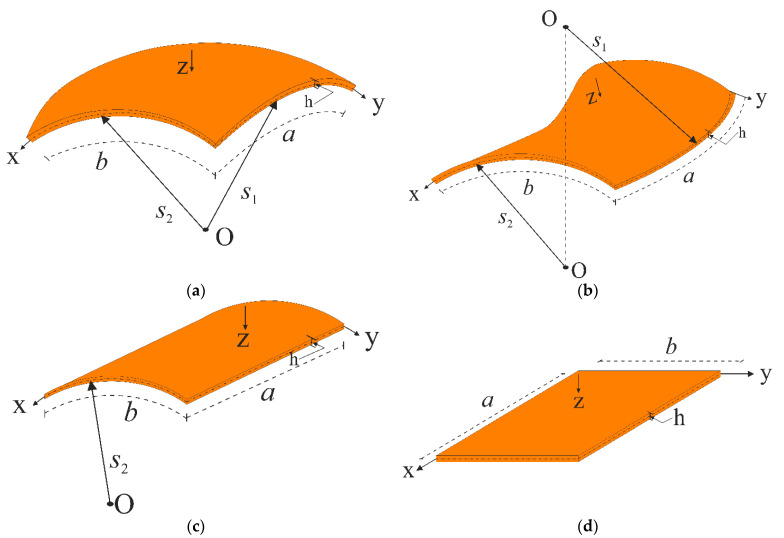
CNT patterned construction elements: (**a**) spherical shell, (**b**) hypar shell, (**c**) cylindrical panel and (**d**) rectangular plate.

While denoting the displacements in the directions of the x and y axes with the symbols u and v, respectively, the displacement in the direction of the z-axis is indicated as w. When the curvatures of the shells in the x and y directions are represented by $k_{1}$ and $k_{2}$, respectively, $k_{1}=\frac{1}{s_{1}}$ and $k_{2}=\frac{1}{s_{2}}$ for a spherical shell, $k_{1}=-\frac{1}{s_{1}}$ and $k_{2}=\frac{1}{s_{2}}$ for a hypar shell, $k_{1}=0$ and $k_{2}=\frac{1}{s_{2}}$ for a cylindrical panel, $k_{1}=k_{2}=0$ for a rectangular plate will be valid. Here, $s_{1}$ and $s_{2}$ are the radii of curvature in the x and y directions, respectively (see Figure 2).

The volume fraction of carbon nanotubes is modelled as a linear function of the thickness coordinate as follows:

(2) $$V_{\bar{z}}^{cnt}=f_{\bar{z}}^{cnt}V_{*}^{cnt}\;\;(\bar{z}=z/h)$$

where $V_{cnt}^{*}$ is the total volume fraction of CNTs and is defined as:

(3) $$V_{cnt}^{*}=\frac{M_{cnt}}{M_{cnt}+\frac{\rho_{cnt}}{\rho_{m}}\left(1-M_{cnt}\right)}$$

$M_{cnt}$ is the mass fraction of CNTs and $f(\bar{z})$ is a continuous function and it is defined as three different linear functions as follows [16]:

(4) $$f_{\bar{z}}^{cnt}=\;\;\left\{ \begin{array}{l} 1+2\bar{z}\\ 1-2\bar{z}\\ \;4\left|\bar{z}\right| \end{array}\right.$$

in which corresponding to the CNT patterns in the matrix, we will denote the case $f_{\bar{z}}^{cnt}=\;\;1+2\bar{z}$ by the symbol V, the case $f_{\bar{z}}^{cnt}=\;\;\;1-2\bar{z}$ by the symbol O and the case $f_{\bar{z}}^{cnt}=\;4\left|\bar{z}\right|$ by the symbol X. In addition, the case $f_{\bar{z}}^{cnt}=\;\;1$ corresponds to the uniform distribution of the CNTs in matrix and is denoted by U.

**Figure 3:**
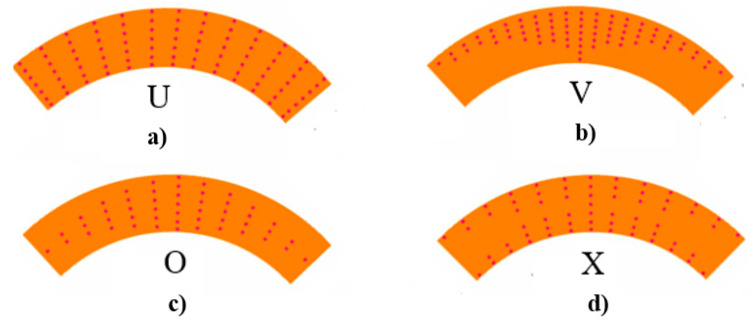
The cross section of CNT patterned construction elements: (**a**) U pattern, (**b**) V pattern, (**c**) O pattern and (**d**) X pattern.

The cross-section of CNT patterned construction elements is illustrated in Figure 3.

The mechanical properties of CNT patterned construction elements are modeled mathematically as the linear functions of the thickness coordinate as follows [16,30]:

(5) $$E_{\bar{z}}^{11}=\mu_{1}V_{\bar{z}}^{cnt}E_{cnt}^{11}+V_{m}E_{m},\;\;\frac{\mu_{2}}{E_{\bar{z}}^{22}}=\frac{V_{\bar{z}}^{cnt}}{E_{cnt}^{22}}+\frac{V_{m}}{E_{m}},\;\;\frac{\mu_{3}}{G_{\bar{z}}^{12}}=\frac{V_{\bar{z}}^{cnt}}{G_{cnt}^{12}}+\frac{V_{m}}{G_{m}},\;\;G_{\bar{z}}^{13}=G_{\bar{z}}^{12},\;\;G_{\bar{z}}^{23}=1.2G_{\bar{z}}^{12}$$

where $\mu_{k}(k=1,2,3)$ indicates the efficiency parameters, $V_{m}$ indicates volume fraction of the matrix, $E_{m}$, $E_{cnt}^{ij}$ and $G_{cnt}^{12}$ indicate the elastic moduli of the matrix and CNTs, respectively. The volume fractions of constituents are related as $V_{\bar{z}}^{cnt}+V_{m}=1$, while Poisson ratio and density of nanocomposites do not depend on the position and are expressed as:

(6) $$\nu^{12}=V_{*}^{cnt}\nu_{cnt}^{11}+V_{m}\nu_{m},\;\;\;\rho =V_{*}^{cnt}\rho_{cnt}+V_{m}\rho_{m}$$

in which is satisfied the following equality:

(7) $$\frac{E_{\bar{z}}^{11}}{\nu^{12}}=\frac{E_{\bar{z}}^{22}}{\nu^{21}}$$

## 3. Basic Relations

The mathematical model of stress–strain relationships for CNT patterned functionally graded construction elements based on the FSDT is expressed as [25,26]:

(8) $$\left[\begin{array}{l} \sigma^{11}\\ \sigma^{22}\\ \sigma^{12} \end{array}\right]=\left[\begin{array}{ccc} Q_{\bar{z}}^{11} & \;Q_{\bar{z}}^{12} & 0\\ Q_{\bar{z}}^{21} & Q_{\bar{z}}^{22} & 0\\ 0 & 0 & Q_{\bar{z}}^{66} \end{array}\right]\;\;\left[\begin{array}{c} e^{11}\\ e^{22}\\ e^{12} \end{array}\right]$$

and

(9) $$\left[\begin{array}{l} \sigma^{13}\\ \sigma^{23} \end{array}\right]=\left[\begin{array}{cc} Q_{\bar{z}}^{55} & 0\\ 0 & Q_{\bar{z}}^{44} \end{array}\right]\;\;\left[\begin{array}{l} e^{13}\\ e^{23} \end{array}\right]$$

where [σ] and [e] are the stress and strain tensors and $Q_{\bar{z}}^{ij}(i=1,2,j=1,2,3)$ are the mechanical characteristics of CNT patterned constructions and are defined as:

(10) $$\begin{array}{l} Q_{\bar{z}}^{11}=\frac{E_{\bar{z}}^{11}}{1-\nu^{12}\nu^{21}},Q_{\bar{z}}^{12}=\frac{\nu^{21}E_{\bar{z}}^{11}}{1-\nu^{12}\nu^{21}}=\frac{\nu^{12}E_{\bar{z}}^{22}}{1-\nu^{12}\nu^{21}}=Q_{\bar{z}}^{21},\;\;Q_{\bar{z}}^{22}=\frac{E_{\bar{z}}^{22}}{1-\nu^{12}\nu^{21}}\\ Q_{\bar{z}}^{44}=G_{\bar{z}}^{23},\;\;\;Q_{\bar{z}}^{55}=G_{\bar{z}}^{13},\;\;\;Q_{\bar{z}}^{66}=G_{\bar{z}}^{12} \end{array}$$

As suggested in Ref. [1], based on the assumptions of the first order shear deformation theory, the transverse shear stresses can be expressed by the rotation angle functions for the normal of the mid-surface, $\psi_{1}(x,y,t)$ and $\psi_{2}(x,y,t)$ as follows:

(11) $$\sigma^{13}=\frac{d\varphi_{z}^{ss}}{dz}\psi_{1}(x,y,t),\;\;\sigma^{23}=\frac{d\varphi_{z}^{ss}}{dz}\psi_{2}(x,y,t)$$

where $\varphi_{z}^{ss}$ indicates the shear strain function and *t* is a time.

In this study, by considering von Kármán kinematic nonlinearity, assumptions (11) and using Donnell-type nonlinear theory, the relationship between strains with displacements and angles of rotation for CNT patterned constructions with double curvature can be constructed as follows [1,36,41]:

(12) $$\begin{array}{l} e^{11}=e_{0}^{11}-z\frac{\partial^{2}w}{\partial x^{2}}+J_{\bar{z}}^{1}\frac{\partial \psi_{1}}{\partial x},\;\;\;\;\;\;e^{22}=e_{0}^{22}-z\frac{\partial^{2}w}{\partial y^{2}}+J_{\bar{z}}^{2}\frac{\partial \psi_{2}}{\partial y}\\ e^{12}=e_{0}^{12}-2z\frac{\partial^{2}w}{\partial x\partial y}+J_{\bar{z}}^{1}\frac{\partial \psi_{1}}{\partial y}+J_{\bar{z}}^{2}\frac{\partial \psi_{2}}{\partial x} \end{array}$$

where

(13) $$e_{0}^{11}=\frac{\partial u}{\partial x}-\frac{w}{s_{1}}+\frac{1}{2}{\left(\frac{\partial w}{\partial x}\right)}^{2},\;\;e_{0}^{22}=\frac{\partial v}{\partial x}-\frac{w}{s_{2}}+\frac{1}{2}{\left(\frac{\partial w}{\partial y}\right)}^{2},\;\;e_{0}^{12}=\frac{\partial w}{\partial x}+\frac{\partial w}{\partial y}+\frac{\partial w}{\partial x}\frac{\partial w}{\partial y}$$

(14) $$J_{z}^{1}=h\int_{0}^{\bar{z}}\frac{1}{G_{\bar{z}}^{13}}\frac{d\varphi_{z}^{ss}}{dz}d\bar{z},\;\;\;\;\;\;\;J_{z}^{2}=h\int_{0}^{\bar{z}}\frac{1}{G_{\bar{z}}^{23}}\frac{d\varphi_{z}^{ss}}{dz}d\bar{z}$$

The forces and moments for CNT patterned construction elements are found from the following integrals [1,41]:

(15) $$\left(T_{kp},\;\;q_{p}\right)=h\int_{-1/2}^{1/2}\left(\sigma_{kp},\sigma_{1p_{1}}\right)d\bar{z},\;\;\;\;M_{kp}=h^{2}\int_{-1/2}^{1/2}\sigma_{kp}\bar{z}d\bar{z}\;\;\;\;(k,p=1,2,\;\;p_{1}=2,3)$$

where in-plane and shear forces are denoted by $T_{kp}$ and $q_{p}$, respectively and moments by $M_{kp}$.

With the Airy stress function, ϕ, in-plane forces are defined as follows [1,41]:

(16) $$T_{11}=h\frac{\partial^{2}\phi }{\partial y^{2}},T_{22}=h\frac{\partial^{2}\phi }{\partial x^{2}},T_{12}=-h\frac{\partial^{2}\phi }{\partial x\partial y}$$

After substituting the Relations (8) and (9) in (15), considering the Equations (12) and (16) in these integrals, we obtain the following expressions for moments, shear forces and strains on the mid-surface:

(17) $$\left[\begin{array}{c} M_{11}\\ M_{22}\\ M_{12} \end{array}\right]=\left[\begin{array}{ccc} m_{11} & m_{12} & 0\\ m_{21} & m_{22} & 0\\ 0 & 0 & m_{31} \end{array}\right]\left[\begin{array}{c} \phi_{,yy}\\ \phi_{,xx}\\ -\phi_{,xy} \end{array}\right]-\left[\begin{array}{ccc} m_{13} & m_{14} & 0\\ m_{23} & m_{24} & 0\\ 0 & 0 & m_{32} \end{array}\right]\left[\begin{array}{c} w_{,xx}\\ w_{,yy}\\ w_{,xy} \end{array}\right]+\left[\begin{array}{ccc} m_{15} & 0 & 0\\ m_{25} & 0 & 0\\ 0 & 0 & m_{35} \end{array}\right]\left[\begin{array}{l} \psi_{1,x}\\ 0\\ \psi_{1,y} \end{array}\right]+\left[\begin{array}{ccc} m_{18} & 0 & 0\\ m_{28} & 0 & 0\\ 0 & 0 & m_{38} \end{array}\right]\left[\begin{array}{l} \psi_{2,y}\\ 0\\ \psi_{2,x} \end{array}\right]$$

(18) $$\left[\begin{array}{l} q_{1}\\ q_{2} \end{array}\right]=\left[\begin{array}{cc} J_{3} & \psi_{1}\\ J_{4} & \psi_{2} \end{array}\right]$$

(19) $$\left[\begin{array}{c} e_{0}^{11}\\ e_{0}^{22}\\ e_{0}^{12} \end{array}\right]=\left[\begin{array}{ccc} n_{11} & n_{12} & 0\\ n_{21} & n_{22} & 0\\ 0 & 0 & n_{31} \end{array}\right]\left[\begin{array}{c} \phi_{,yy}\\ \phi_{,xx}\\ -\phi_{,xy} \end{array}\right]-\left[\begin{array}{ccc} n_{13} & n_{14} & 0\\ n_{23} & n_{24} & 0\\ 0 & 0 & n_{32} \end{array}\right]\left[\begin{array}{c} w_{,xx}\\ w_{,yy}\\ w_{,xy} \end{array}\right]+\left[\begin{array}{ccc} n_{15} & 0 & 0\\ n_{25} & 0 & 0\\ 0 & 0 & -n_{35} \end{array}\right]\left[\begin{array}{l} \psi_{1,x}\\ 0\\ \psi_{1,y} \end{array}\right]+\left[\begin{array}{ccc} n_{18} & 0 & 0\\ n_{28} & 0 & 0\\ 0 & 0 & -n_{38} \end{array}\right]\left[\begin{array}{l} \psi_{2,y}\\ 0\\ \psi_{2,x} \end{array}\right]$$

where the comma indicates the partial derivative with respect to the appropriate coordinates, $m_{kp},n_{kp}(k=1,2,3,p=1,2,\ldots ,8)$ and $J_{k}(k=3,4)$ are described in Appendix A (Equations (A1) and (A2)).

## 4. Basic Equations and Solution

The nonlinear deformation compatibility equation of construction elements with double curvature is expressed as follows [41]:

(20) $$\frac{\partial^{2}e_{0}^{11}}{\partial y^{2}}+\frac{\partial^{2}e_{0}^{22}}{\partial x^{2}}-\frac{\partial^{2}e_{0}^{12}}{\partial x\partial y}={\left(\frac{\partial^{2}w}{\partial x\partial y}\right)}^{2}-\frac{\partial^{2}w}{\partial x^{2}}\frac{\partial^{2}w}{\partial y^{2}}-\left(\frac{1}{s_{2}}\frac{\partial^{2}w}{\partial x^{2}}+\frac{1}{s_{1}}\frac{\partial^{2}w}{\partial y^{2}}\right)$$

To derive the nonlinear deformation compatibility equation for the CNT patterned constructions, the Equation (19) is substituted in the Equation (20), and after some operations it transforms into the following form:

(21) $$\begin{array}{l} h\left[n_{11}\frac{\partial^{4}\phi }{\partial y^{4}}+(n_{12}+n_{21}+n_{31})\frac{\partial^{4}\phi }{\partial x^{2}\partial y^{2}}+n_{22}\frac{\partial^{4}\phi }{\partial x^{4}}\right]-n_{23}\frac{\partial^{4}w}{\partial x^{4}}-\left(n_{24}+n_{13}-n_{32}\right)\frac{\partial^{4}w}{\partial x^{2}\partial y^{2}}\\ -n_{14}\frac{\partial^{4}}{\partial y^{4}}+\left(\frac{1}{s_{2}}\frac{\partial^{2}w}{\partial x^{2}}+\frac{1}{s_{1}}\frac{\partial^{2}w}{\partial y^{2}}\right)-{\left(\frac{\partial^{2}w}{\partial x\partial y}\right)}^{2}+\frac{\partial^{2}w}{\partial x^{2}}\frac{\partial^{2}w}{\partial y^{2}}\\ +n_{25}\frac{\partial^{3}\psi_{1}}{\partial x^{3}}+n_{15}\frac{\partial^{3}\psi_{1}}{\partial x\partial y^{2}}+n_{35}\frac{\partial^{3}\psi_{1}}{\partial x\partial y^{2}}+n_{28}\frac{\partial^{3}\psi_{2}}{\partial x^{2}\partial y}+n_{38}\frac{\partial^{3}\psi_{2}}{\partial x^{2}\partial y}+n_{18}\frac{\partial^{3}\psi_{2}}{\partial y^{3}}=0 \end{array}$$

The Equation (21) will be solved in the following simply supported boundary conditions [41]:

(22) $$\begin{array}{l} w=M_{11}=\psi_{2}=\;\frac{\partial^{2}\phi }{\partial y^{2}}=0,\;\;\mathrm{when}\;\;x=0\;\;\mathrm{and}\;\;a\\ w=M_{22}=\psi_{1}=\;\frac{\partial^{2}\phi }{\partial x^{2}}=0,\;\;\mathrm{when}\;\;y=0\;\;\mathrm{and}\;\;b \end{array}$$

The functions $w,\;\;\psi_{1},\;\;\psi_{2}$ that satisfy the boundary conditions (22) are sought as follows [25]:

(23) $$w=w_{0}(t)\;sin(i_{1}x)\;sin(j_{1}y),\;\;\psi_{1}=\psi_{01}(t)\;cos(i_{1}x)\;sin(j_{1}y),\;\;\psi_{2}=\psi_{0}{}_{2}(t)\;sin(j_{1}x)\;cos(j_{1}y)$$

where $w_{0}(t)$ and $\psi_{0k}(t)\;\;(k=1,2)$ are functions of the time and $i_{1}=\frac{i\pi }{a},\;\;\;\;j_{1}=\frac{j\pi }{b}$, in which (i, j) is vibrational mode in the x and y directions.

Substituting (23) into Equation (21), the following expression is found for the ϕ from a particular solution of the nonhomogeneous differential equation:

(24) $$\phi =\theta_{1}\cos(2i_{1}x)+\theta_{2}\cos(2j_{1}y)+\theta_{3}\sin(i_{1}x)\sin(j_{1}y)$$

where the following definitions apply:

(25) $$\theta_{1}=\frac{\delta_{14}w_{0}^{2}}{32i_{1}^{4}\delta_{3}},\;\;\;\theta_{2}=\frac{\delta_{14}w_{0}^{2}}{32j_{1}^{4}\delta_{1}},\;\;\;\;\theta_{3}=\frac{\delta_{11}w_{0}+\delta_{12}\psi_{01}+\delta_{13}\psi_{02}}{\delta_{1}j_{1}^{4}+\delta_{2}i_{1}^{2}j_{1}^{2}+\delta_{3}i_{1}^{4}}$$

in which

(26) $$\begin{array}{l} \delta_{1}=n_{11}h,\;\;\;\delta_{2}=(n_{12}+n_{21}+n_{31})h,\;\;\;\delta_{3}=n_{22}h,\;\;\\ \delta_{11}=n_{23}i_{1}^{4}+(n_{24}+n_{13}-n_{32})i_{1}^{2}j_{1}^{2}+n_{14}j_{1}^{4}+\frac{i_{1}^{2}}{s_{2}}+\frac{j_{1}^{2}}{s_{1}},\\ \delta_{12}=-n_{25}i_{1}^{3}-(n_{15}+n_{35})i_{1}j_{1}^{2},\;\;\;\delta_{13}=-(n_{28}+n_{38})i_{1}^{2}j_{1}-n_{18}j_{1}^{3},\;\;\;\delta_{14}=i_{1}^{2}j_{1}^{2} \end{array}$$

The mathematical model of nonlinear motion equations of double-curved construction elements is expressed as follows [41]:

(27) $$\begin{array}{l} \frac{\partial M_{11}}{\partial x}+\frac{\partial M_{12}}{\partial y}-q_{1}+\rho_{1}\frac{\partial^{3}w}{\partial x\partial t^{2}}-\rho_{2}\frac{\partial^{2}\psi_{1}}{\;\partial t^{2}}=0\\ \frac{\partial M_{21}}{\partial x}+\frac{\partial M_{22}}{\partial y}-q_{2}+\rho_{1}\frac{\partial^{3}w}{\partial y\partial t^{2}}-\rho_{3}\frac{\partial^{2}\psi_{2}}{\;\partial t^{2}}=0\\ \frac{\partial q_{1}}{\partial x}+\frac{\partial q_{2}}{\partial y}+\frac{T_{11}}{s_{1}}+\frac{T_{22}}{s_{2}}+T_{11}\frac{\partial^{2}w}{\;\partial x^{2}}+2T_{12}\frac{\partial^{2}w}{\partial x\partial y}+T_{22}\frac{\partial^{2}w}{\;\partial y^{2}}=\rho h\frac{\partial^{2}w}{\partial t^{2}} \end{array}$$

where $\rho_{k}(k=1,2,3)$ are coefficients of the normal and rotary inertia and are defined as:

(28) $$\;\rho_{1}=\rho \int_{-h/2}^{h/2}\;z^{2}dz,\;\;\;\rho_{2}=\rho \int_{-h/2}^{h/2}\;zJ_{z}^{1}dz,\;\;\;\;\rho_{3}=\rho \int_{-h/2}^{h/2}\;zJ_{z}^{2}dz$$

To derive the nonlinear dynamic equation of nanocomposite construction members with double curvature, the Equations (16)–(18) are substituted in the set of Equation (27), and after some mathematical operations, it transforms into the following form:

(29) $$\begin{array}{l} L_{11}(\phi )+L_{12}(w)+L_{13}(\psi_{1})+L_{14}(\psi_{2})=0\\ L_{21}(\phi )+L_{22}(w)+L_{23}(\psi_{1})+L_{24}(\psi_{2})=0\\ L_{31}(\phi )+L_{32}(w)+L_{33}(\psi_{1})+L_{34}(\psi_{2})+L_{35}(\phi ,w)=0 \end{array}$$

where the nonlinear differential operators $L_{kp}(k=1,2,3,p=1,2,\ldots ,5)$ are given in Appendix A (Equation (A3)).

By applying the Galerkin method to the system of partial differential Equation (29), after integration, the following set of nonlinear ordinary differential equations is obtained:

(30) $$\begin{array}{l} c_{11}w_{0}+c_{11}^{nl}w_{0}{}^{2}+c_{11}^{t}\frac{d^{2}w_{0}}{dt^{2}}+c_{12}\psi_{01}+c_{12}^{t}\frac{d^{2}\psi_{01}}{dt^{2}}+c_{13}\psi_{02}=0,\\ c_{21}w_{0}+c_{21}^{nl}w_{0}{}^{2}+c_{21}^{t}\frac{d^{2}w_{0}}{dt^{2}}+c_{22}\psi_{01}+c_{23}\psi_{02}+c_{23}^{t}\frac{d^{2}\psi_{02}}{dt^{2}}=0,\\ \rho h\frac{d^{2}w_{0}}{dt^{2}}+c_{31}w_{0}+c_{31}^{NL}w_{0}{}^{2}+c_{32}w_{0}{}^{3}+c_{33}\psi_{01}+c_{34}\psi_{02}=0 \end{array}$$

where $c_{kp}(k=1,2,3,p=1,2,3,4)$ are coefficients depending on the properties of nanocomposite structural members with double curvature and are given in Appendix A (Equations (A4) and (A5)).

Due to the smallness of the inertia terms with the upper index *t*, ignoring these terms in Equation (30), and eliminating the functions $\psi_{01}$ and $\psi_{02}$ from the obtained equations, it is transformed into the following nonlinear ordinary differential equation with quadratic and cubic nonlinearities:

(31) $$\frac{d^{2}w_{0}}{dt^{2}}+{\left(\Omega_{sdt}^{nf}\right)}^{2}w_{0}+u_{1}w_{0}{}^{2}+u_{2}w_{0}{}^{3}=0$$

where $\Omega_{sdt}^{nf}$ is the linear or natural frequency for CNT patterned construction members with double curvature in the scope of FSDT and defined as:

(32) $$\Omega_{sdt}^{nf}=\sqrt{\frac{c_{31}^{*}}{\left(V_{*}^{cnt}\rho_{cnt}+V_{m}\rho_{m}\right)h}}$$

where

(33) $$\begin{array}{l} u_{1}=\frac{c_{31}^{*nl}}{\left(V_{*}^{cnt}\rho_{cnt}+V_{m}\rho_{m}\right)h},\;\;\;u_{2}=\frac{c_{32}}{\left(V_{*}^{cnt}\rho_{cnt}+V_{m}\rho_{m}\right)h},\;\;\\ c_{31}^{*}=c_{31}-\frac{c_{21}c_{34}}{c_{23}}+\left(c_{33}-\frac{c_{22}c_{34}}{c_{23}}\right)\frac{c_{11}c_{23}-c_{21}c_{13}}{c_{22}c_{13}-c_{23}c_{12}}\\ c_{31}^{*nl}=c_{31}^{nl}-\frac{c_{34}c_{21}^{nl}}{c_{23}}+\left(\frac{c_{34}c_{22}}{c_{23}}-c_{33}\right)\frac{c_{11}^{nl}c_{23}-c_{13}c_{21}^{nl}}{c_{12}c_{23}-c_{13}c_{22}} \end{array}$$

The initial conditions are described as:

(34) $$w_{0}={\bar{w}}_{0},\;\;\;\frac{dw_{0}}{dt}=0\;\;\;\mathrm{as}\;\;\;\;\;t=0$$

In the current study, the Grigolyuk method is applied to the solution of Equation (31). The Grigolyuk method is a variational method [42]. This method is used to solve the nonlinear ordinary differential equation. To determine the nonlinear frequency-amplitude dependence for nanocomposite construction members with double curvature patterned by CNTs within the FSDT, each part of Equation (31) is multiplied by cos(Ωt) and integrated in one quarter of the period (from 0 to T/4), satisfying the orthogonality condition:

(35) $$\int_{0}^{T/4}\left[\frac{d^{2}w_{0}}{dt^{2}}+{\left(\Omega_{sdt}^{nf}\right)}^{2}w_{0}+u_{1}w_{0}{}^{2}+u_{2}w_{0}{}^{3}\right]\cos(\Omega t)dt=0$$

where, the symbol T=2π/Ω indicates the large amplitude vibration period and $\Omega =\Omega_{sdt}^{nl}$ indicates the nonlinear frequency of CNT patterned nanocomposite construction elements with double curvature in the framework of SDT.

When the expression of T=2π/Ω is substituted at the upper limit of the integral (35), it turns into the following equation:

(36) $$\int_{0}^{\pi /2\Omega }\left[\frac{d^{2}w_{0}}{dt^{2}}+{\left(\Omega_{sdt}^{nf}\right)}^{2}w_{0}+u_{1}w_{0}{}^{2}+u_{2}w_{0}{}^{3}\right]\cos\left(\Omega t\right)dt=0$$

The trial function is expressed as follows:

(37) $$w_{0}(t)={\bar{w}}_{0}\cos\left(\Omega t\right)$$

where ${\bar{w}}_{0}=w_{\max}$ is the maximum amplitude of the displacement w.

Substituting (37) into (36), after integrating, for the amplitude-frequency characteristics of the nonlinear free vibration, the following dependence is obtained:

(38) $$\Omega_{sdt}^{nl}={\left[{\left(\Omega_{sdt}^{nf}\right)}^{2}+\frac{8{\bar{u}}_{1}}{3\pi }\frac{A}{h}+\frac{3{\bar{u}}_{2}}{4}{\left(\frac{A}{h}\right)}^{2}\right]}^{1/2}$$

where ${\bar{u}}_{1}=u_{1}h$ and ${\bar{u}}_{2}=u_{2}h^{2}$.

The following dependence is used for the ratio of nonlinear frequency to linear frequency for CNT patterned construction elements with double curvature:

(39) $$\frac{\Omega_{sdt}^{nl}}{\Omega_{sdt}^{nf}}={\left[1+\frac{8{\bar{u}}_{1}}{3\pi {\left(\Omega_{sdt}^{nf}\right)}^{2}}\frac{A}{h}+\frac{3{\bar{u}}_{2}}{4{\left(\Omega_{sdt}^{nf}\right)}^{2}}{\left(\frac{A}{h}\right)}^{2}\right]}^{1/2}$$

The Equations (32), (38) and (39) are transformed expressions for linear and nonlinear frequencies and their ratios, respectively, within the CST when transverse shear deformations are eliminated from the basic relations and equations of CNT patterned construction elements with double curvature.

## 5. Discussion

### 5.1. Comparative Studies

In this section, the accuracy of analytical formulas obtained for frequencies in our study is checked by comparing them with other results in the literature.

The magnitudes of dimensionless frequency parameter, $\Omega_{1sdt}^{nf}=\Omega_{sdt}^{nf}\frac{a^{2}}{h}{\left(\frac{\rho_{m}}{E_{m}}\right)}^{1/2}\;$ for construction elements such as spherical and hypar shells, cylindrical panels and plates patterned by CNTs are compared with the results of Pouresmaeeli and Fazelzadeh [17] with (i, j)=(1,1), a=20h,   b=20h, $a/s_{1}=0.5$ and tabulated in Table 1. The Equation (32) is applied to obtain the linear frequency values that are used in this comparison. The mechanical properties of constructions made of the poly methyl methacrylate (PMMA) used as a matrix and CNTs used as additives, respectively, are as follows: $E_{m}=2.1\;\times 10^{9}\;\mathrm{Pa}$, $\nu_{m}=0.34$, $\rho_{m}=1.15\times 10^{3}\;\mathrm{kg}/m^{3}$ and $E_{cnt}^{11}=5.6466\;\times \;10^{12}\;\mathrm{Pa},$$E_{cnt}^{22}=7.08\times 10^{12}\;\mathrm{Pa},$ $G_{cnt}^{12}=1.9445\times 10^{12}\;\mathrm{Pa},$ $\;\nu_{cnt}^{12}=0.175$, $\;\rho_{cnt}=1.4\times 10^{3}\;\mathrm{kg}/m^{3}$. The efficiency parameters of CNTs in the constructions are taken from Ref. [17]: $\mu_{1}=0.149,\;\;\mu_{2}=\mu_{3}=0.934$ for $V_{*}^{cnt}=0.11$, $\mu_{1}=0.15,\;\;\mu_{2}=\;\mu_{3}=0.941\;$ for $V_{*}^{cnt}=0.14$ and $\mu_{1}=0.149,\;\;\mu_{2}=\mu_{3}=1.381$ for $V_{*}^{cnt}=0.17$. It is seen that the magnitudes of dimensionless linear frequency presented for different construction elements patterned by CNTs in Table 1 are in very good agreement.

In the second example, our results are compared with those of Alijani et al. [43] and Bich et al. [44] for dimensionless linear frequency parameters of homogeneous isotropic construction elements in the framework of SDTs (see Table 2). The Equation (32) is applied to obtain the magnitudes of linear frequency that are used in this comparison. The mechanical properties of constructions are as follows: $E^{11}=E^{22}=E_{m}=7\;\times \;10^{10}\;\mathrm{Pa},\;\nu^{12}=\nu_{m}=0.3177,\;$$\rho_{m}=2702\;\mathrm{kg}/m^{3}$ a=10h,  b=10h. Table 2 is an indication that our results are in good agreement with the results of Refs. [43,44].

### 5.2. New Numerical Analyses and Interpretations

In this subsection, unique numerical analyses and interpretations for different volume fractions, geometric characteristics and vibration modes are presented to examine the nonlinear vibration behavior of CNT patterned homogenous and inhomogeneous constructions, such as plate, panel, spherical and hypar shells in the framework of CST and SDT. The Equations (38) and (39) are used to calculate the magnitudes of the nonlinear frequency and the ratio of the nonlinear frequency to the linear frequency. In the figures, the abbreviations for classical theory and shear deformation theory are shown as (cst) and (sdt), respectively. The shear stresses function constructions with CNT patterns are used as $\varphi_{z,z}^{ss}=1-4{\bar{z}}^{2}$ [1]. A negative sign in ratios means that the frequency values in functionally graded distributions are less than the frequency values in the uniform distribution. The following expressions are used for percentages of the effects of inhomogeneity and shear strains on frequencies: $\frac{\Omega_{HT}-\Omega_{U}}{\Omega_{U}}\times 100\%$ and $\frac{\Omega_{cst}-\Omega_{sdt}}{\Omega_{cst}}\times 100\%$. In the numerical analysis, while, $E_{m}=2.5\;\times 10^{9}\mathrm{Pa},\;\;\nu_{m}=0.34,\;\;\rho_{m}=1150\;\mathrm{kg}/m^{3}$ and $E_{cnt}^{11}=5.6466\;\times \;10^{12}\mathrm{Pa},$ $E_{cnt}^{22}=7.08\times 10^{12}\mathrm{Pa},\;$$G_{cnt}^{12}=1.9445\times 10^{12}\;\mathrm{Pa},\;\;\;\nu_{cnt}^{12}=0.175$, $\;\rho_{cnt}=1400\;\mathrm{kg}/m^{3}$ are used for the mechanical properties of PMMA and CNTs, respectively; the data for the total volume fractions and efficiency parameters of CNTs are presented in Table 3 [16].

**Figure 4:**
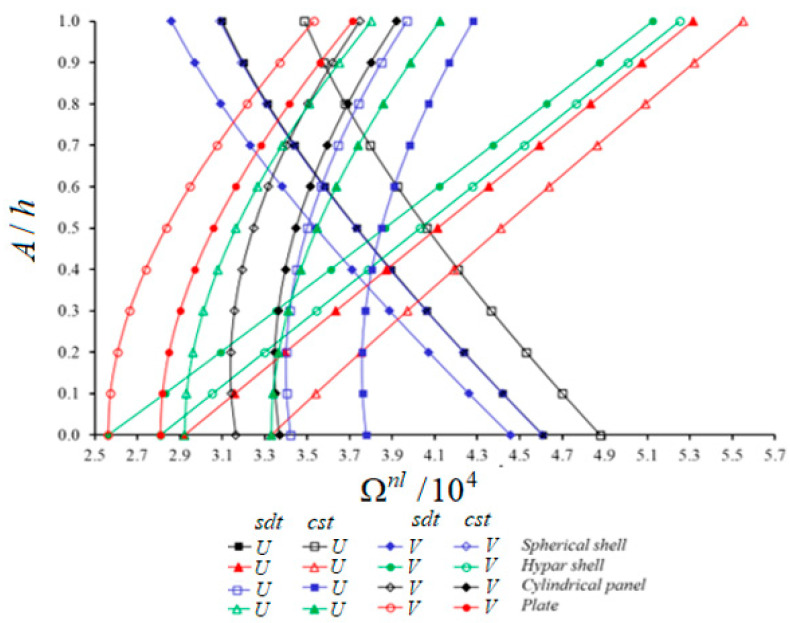
Variation of NLFVs for spherical shell, hypar shell, cylindrical panel and plate patterned by CNTs within CST and SDT against the A/h.

Figure 4 shows the variation of nonlinear frequency values (NLFVs) for the spherical and hypar shells, cylindrical panel and plate patterned by CNTs against the A/h. The analysis uses the constructions with U and V patterns, taking into account the following data: $V_{*}^{cnt}=0.12,\;s_{1}/a\;=\;1,\;a=b=20h,\;\left(i,\;j\right)=\left(1,1\right)$. Depending on the increase of A/h, the NLFVs for plates and hypar shells increase while those for spherical shells with U and V patterns decrease. The NLFVs of cylindrical panels first decrease, and after reaching the minimum value, they increase. The shear strains effect on NLFVs for composite constructions with the V pattern is smaller than for composite constructions with the U pattern. For example, starting from the highest, the shear strains effect difference between U and V patterns in percentage is: (3.68%), (3.56%), (3.56%) and (3.34%) for the spherical shell, hypar shell, plate and cylindrical panel, respectively. Due to the increase of A/h, the V pattern effect on the NLFVs of the plate and hypar shell decreases, while this effect increases for the spherical shell, and for the cylindrical panel the V pattern effect first increases and then decreases within SDT. For example, depending on the rise of A/h from 0 to 1, the influence of the V pattern on NLFVs for the hypar shell (8.68%) and the plate (5.15%) decreases, while for the spherical shell it increases (4.39%) within the SDT. Likewise, this effect first increases slightly (about 0.1%) and then decreases (1.89%) for the cylindrical panel within the SDT. The transverse shear strains reduce the V pattern effect on NLFVs compared to the CST. The largest V pattern effect difference between CST and SDT appears in the spherical shell (3.67%), in the hypar shell and plate (3.42%), while in the cylindrical panel (3.29%).

**Figure 5:**
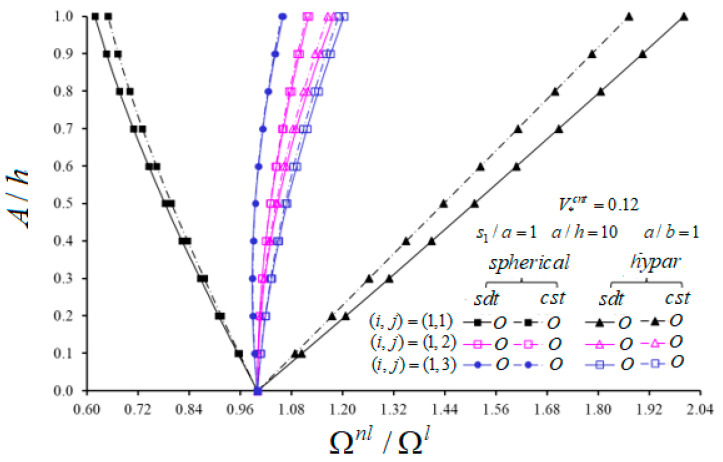
Variation of nonlinear frequency to linear frequency ratio of spherical and hypar shells with O pattern in the framework of CST and SDT against the A/h for (1, *j*) and $V_{*}^{cnt}=0.12$.

**Figure 6:**
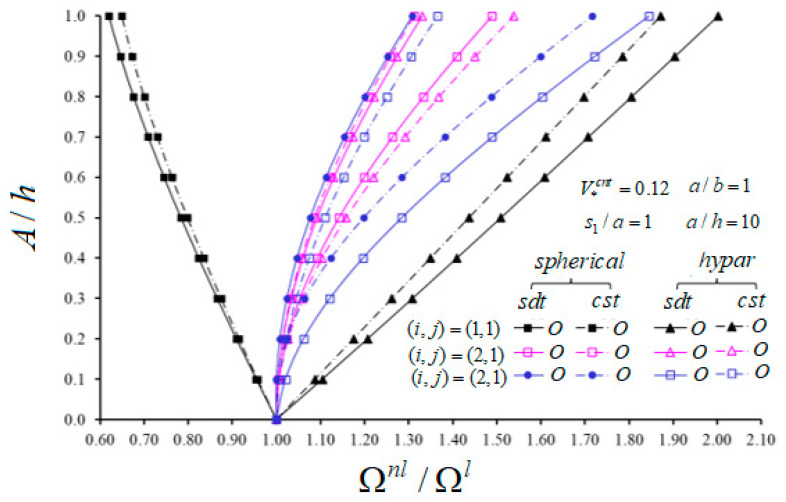
Variation of nonlinear frequency to linear frequency ratio of spherical and hypar shells with O pattern in the framework of CST and SDT against the A/h for (*i*, 1) and $V_{*}^{cnt}=0.12$.

The change of nonlinear frequency to linear frequency ratio of spherical and hypar shells with O patterns in the framework CST and SDT against the A/h for different vibration modes (i,j) and $V_{*}^{cnt}=0.12$ are presented in Figure 5 and Figure 6. Other parametric data are given in figures. As can be seen from Figure 5 and Figure 6, the effect of nonlinearity in hypar shells with the O pattern increases depending on the increase of A/h for all modes (i,j), while that of spherical shells with the O pattern decreases for the mode (i,j)=(1,1). Additionally, the influences of nonlinearity of spherical shells with the O pattern decrease in the range of A/h≤0.3 for mode (i,j)=(1,3); it increases in other cases. Depending on the passes of n from 1 to 2, the influence of nonlinearity in the spherical shell increases, while that in the hypar shells with the O pattern decreases. As the wave number j passes from 2 to 3, it arises vice versa from previous cases. Likewise, as the wave number i passes from 1 to 2, the influence of nonlinearity in spherical shells increases, whereas in hypar shells it decreases. As the wave number i passes from 2 to 3, the influence of nonlinearity increases in hypar shells, while it decreases in spherical shells with the O pattern for A/h≤0.2 and then increases for A/h>0.2. When both shallow shells with the O pattern are compared with each other, the influences of nonlinearity in hypar shells are more considerable than those of spherical shells. While the nonlinear frequency to linear frequency difference between shallow shells with the O pattern increases depending on the increase of A/h, it first decreases and takes its lowest value and then increases continuously due to the increase of wave numbers.

The influence of geometrical nonlinearity in spherical shells within CST is greater than that within SDT; in all other cases, it is vice versa, as (i,j)=(1,1). When both shallow shells with the O pattern are compared, the shear strains effect on the nonlinear frequency to linear frequency ratios for hypar shells is greater than that of spherical shells. With the increase of A/h from 0 to 1, the shear strains effect differences between spherical and hypar shallow shells increase except for modes (i,j)=(1,1) and (i,j)=(3,1). The largest shear strains effect differences between spherical and hypar shallow shells are (3.15%), (0.66%), (0.81%), (2.15%) and (4.51%) for modes (i,j)=(1,1), (1,2), (1,3), (2,1) and (2,3), respectively.

**Figure 7:**
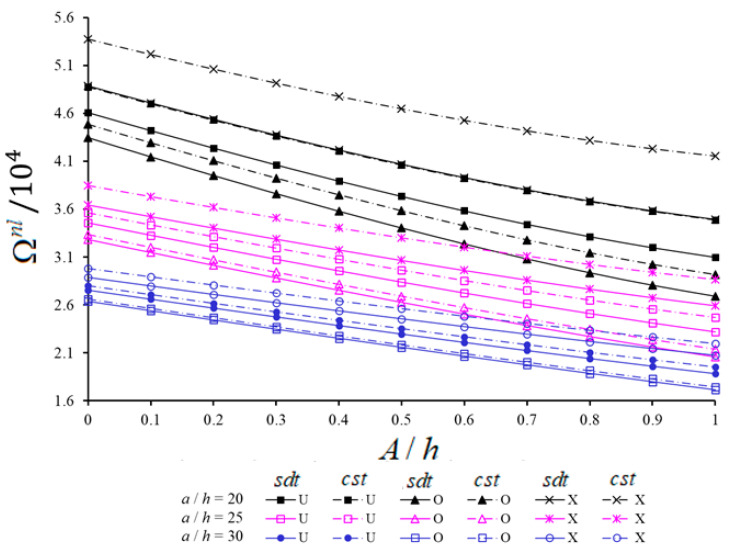
Variation of NLFVs of spherical shells with U, V, O and X patterns within CST and SDT against the A/h for different a/h.

**Figure 8:**
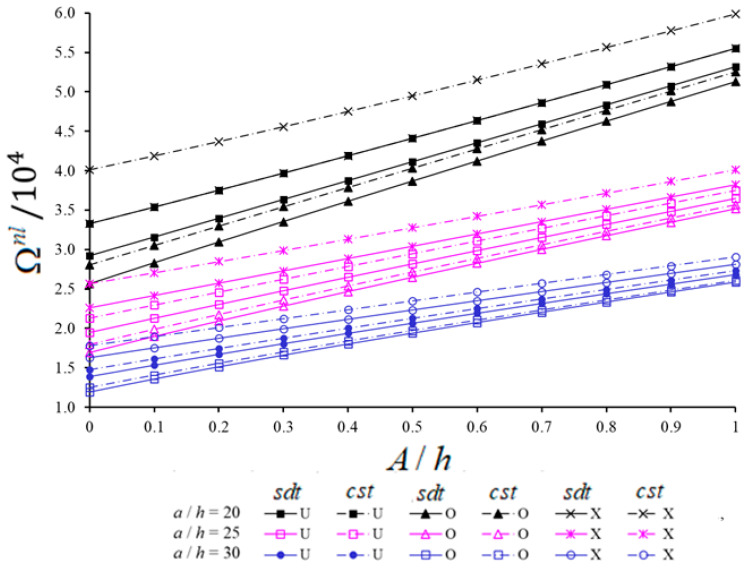
Variations of NLFVs of hypar shells with U, V, O and X patterns within CST and SDT against the A/h for different a/h.

The variations of NLFVs of spherical and hypar shells with U, V, O and X patterns within CST and SDT against the A/h for different a/h are presented in Figure 7 and Figure 8, respectively. Analyses are performed for $V_{*}^{cnt}=0.12$, a/h=b/h=20, 25, 30, $s_{1}/a=1$ and (i,j)=(1,1). With the increase of A/h from 0 to 1, the NLFVs of CNT patterned hypar shells increase, while those of CNT patterned spherical shells decrease. For fixed values of A/h, with the increase of a/h from 20 to 30, the NLFVs of both shells with CNT patterns decrease. When the NLFVs for shallow shells with CNT patterns are compared to each other, the NLFVs of CNT patterned spherical shells in the range of A/h<0.5 are greater than those of hypar shells for fixed a/h. While the NLFVs difference between CNT patterned shallow shells first decreases and then increases with the increase of A/h for fixed a/h, this difference decreases with the increase of a/h for fixed A/h.

With the increase of A/h, the shear strains effect on NLFVs increases in CNT patterned spherical shells, whereas it decreases in CNT patterned hypar shells. For instance, the influences of shear strains on the NLFVs increases from (9.13%) to (15.87%) for spherical shells and decreases from (17.1%) to (7.3%) for hypar shells with the X pattern, as the A/h increases from 0 to 1, at a/h=20. Although the shear strains effect on NLFVs for both shallow shells decreases when the a/h ratio increases, this decrease is more pronounced in CNT patterned spherical shells. For example, as the a/h increases from 20 to 30 for fixed A/h (=0.8), the shear strains effects decrease from (9.93%) to (2.97%) and from (5.06%) to (2.1%) for the U, from (6.47%) to (1.72%) and from (2.95%) to (1.16%) for the V, and from (6.59%) to (1.65%) and from (2.92%) to (1.15%) for the O patterned spherical and hypar shells, respectively.

With the increase of A/h, the effect of CNT patterns on NLFVs for spherical shells decreases, while for hypar shells it increases for both shell theories. For instance, a/h=30 and depending on the increase of A/h from 0 to 1, the O pattern effect on the NLFVs for spherical shells increases from (−4.14%) to (−9.14%) within SDT and from (−4.92%) to (−10.5%) within CST. The O pattern effect on NLFVs for hypar shells decreases from (−13.96%) to (−3.54%) within SDT and from (−15.67%) to (−4.33%) within CST.

With the increase of a/h, the V pattern effect on NLFVs in spherical shells decreases in the framework of both shell theories, while the V pattern effect in hypar shells increases within SDT and decreases in the framework of CST. For example, with the increase of a/h from 20 to 30, the effect of V pattern on NLFVs in spherical shells decreases from (−3.95%) to (−2.4%) within SDT and from (−6.5%) to (−3.23%) within CST at A/h=0.2. In the X pattern spherical shells, it decreases from (7.05%) to (5.54%) and from (11.75%) to (7.26%) within SDT and CST, respectively. Likewise, in the V pattern hypar shells, while the V effect on NLFVs increases from (−8.92%) to (−9.43%) within SDT, it decreases from (−12.11%) to (−10.96%) within CST. In the X pattern hypar shells, while the X effect on NLFVs decreases from (16.46%) to (15.09%), within CST, it increases from (10.46%) to (12.01%) within SDT.

## 6. Conclusions

In this paper, the large amplitude vibration behavior of CNT patterned double-curved construction elements is studied. First, the large amplitude basic relations and dynamic equations are derived in the framework of FSDT. Then, using the Galerkin method, the problem is reduced to the nonlinear vibration of nanocomposite continuous systems with quadratic and cubic nonlinearities. By applying Grigolyuk method to the obtained nonlinear differential equation, the nonlinear frequency-amplitude dependence is obtained. The expressions for nonlinear frequencies of inhomogeneous nanocomposite construction members such as plates, panels and spherical and hypar shells within shear deformation and classical shell theories are found from these expressions in special cases. The accuracy of the results of the current study has been confirmed by comparing them with the reliable results reported in the literature. The effects of nonlinearity, CNT patterns and volume fraction on frequencies are analyzed in terms of quality and quantity within the framework of shear deformation and classical theories.

## Figures and Tables

**Table 1:** Comparison eigenvalues for CNT patterned construction elements with the results of Ref. [17].

Construction Elements	$V_{cn}^{*(1)}$	$\Omega_{1sdt}^{nf}=\Omega_{sdt}^{nf}\frac{a^{2}}{h}{\left(\frac{\rho_{m}}{E_{m}}\right)}^{1/2}\;$					
		U		V		X	
		Ref. [17]	Present Study	Ref. [17]	Present Study	Ref. [17]	Present Study
Spherical shells	0.11	20.238	20.286	18.543	18.685	22.432	22.493
	0.14	21.655	21.756	19.779	19.966	23.997	24.064
	0.17	25.021	25.158	22.951	23.165	27.883	27.893
Hypar shells	0.11	17.106	17.332	14.809	15.114	19.588	19.853
	0.14	18.626	18.924	16.181	16.544	21.225	21.512
	0.17	21.093	21.423	18.225	18.645	24.274	24.524
Cylindrical panels	0.11	18.126	18.116	16.060	16.150	20.548	20.545
	0.14	19.628	19.670	17.391	17.524	22.179	22.178
	0.17	22.380	22.415	19.799	19.949	25.488	25.408
Rectangular Plates	0.11	18.008	17.332	15.701	15.113	20.624	19.853
	0.14	19.608	18.924	17.147	16.544	22.349	21.512
	0.17	22.207	21.424	19.315	18.645	25.557	24.524

**Table 2:** Comparison of dimensionless linear frequency parameters of homogeneous isotropic construction elements within SDTs.

Construction Elements	$s_{1}/a$	$s_{1}/b$	$\Omega_{1sdt}^{nf}=\Omega_{sdt}^{nf}h{\left(\frac{\rho_{m}}{E_{m}}\right)}^{1/2}\;$		
			Ref. [43].	Ref. [44].	Present Study
Spherical shell	2	2	0.0779	0.0767	0.0769
Rectangular plate	∞	∞	0.0597	0.0581	0.0584
Cylindrical panel	∞	2	0.0648	0.0632	0.0636

**Table 3:** Volume fractions and efficiency parameters of the CNTs.

$V_{*}^{cnt}$	$\mu_{1}$	$\mu_{2}$	$\mu_{3}$
0.12	0.137	1.022	0.715
0.17	0.142	1.626	1.138
0.28	0.141	1.585	1.109

## Data Availability

No data were reported in the study.

## Author Agreement

We confirm that this is an original paper, has not been submitted elsewhere for publication and has not been published elsewhere.

## Appendix A

Here $m_{kp},n_{kp}(k=1,2,3,p=1,2,\ldots ,8)$ and $J_{k}(k=3,4)$ are defined as,

(A1) $$\begin{array}{l} m_{11}=a_{111}n_{11}+a_{121}n_{21},\;\;m_{12}=a_{111}n_{12}+a_{121}n_{11},\;\;m_{13}=a_{111}n_{13}+a_{121}n_{23}+a_{112},\;\\ m_{14}=a_{111}n_{14}+a_{121}n_{24}+a_{122},\;\;m_{15}=a_{111}n_{15}+a_{121}n_{25}+a_{151},\;\;m_{18}=a_{111}n_{18}+a_{121}n_{28}+a_{181},\;\\ m_{21}=a_{211}n_{11}+a_{221}n_{21},\;\;\;\;m_{22}=a_{211}n_{12}+a_{221}n_{22},\;\;\;m_{23}=a_{211}n_{13}+a_{221}n_{23}+a_{212},\;\;\\ m_{24}=a_{211}n_{14}+a_{221}n_{24}+a_{222},\;\;\;\;m_{25}=a_{211}n_{15}+a_{221}n_{25}+a_{251},\;\;\;\;\;m_{28}=a_{211}n_{18}+a_{221}n_{28}+a_{281},\;\\ m_{31}=a_{661}n_{35},\;\;m_{32}=a_{661}n_{32}+2a_{662},\;m_{35}=a_{351}-a_{661}n_{35},\;\;\;\;m_{38}=a_{381}-a_{661}n_{38},\;\\ J_{k}=\int_{-h/2}^{h/2}\frac{d\varphi_{z}^{sd}}{dz}dz,\;\;(k=3,4) \end{array}$$

where

(A2) $$\begin{array}{l} n_{11}=\frac{a_{220}}{\Delta },\;\;\;n_{12}=-\frac{a_{120}}{\Delta },\;\;\;n_{13}=\frac{a_{120}a_{211}-a_{111}a_{220}}{\Delta },\;\;\;\;n_{14}=\frac{a_{120}a_{211}-a_{121}a_{220}}{\Delta },\;\\ n_{15}=\frac{a_{250}a_{120}-a_{150}a_{220}}{\Delta },n_{18}=\frac{a_{280}a_{120}-a_{180}a_{220}}{\Delta },\;\;\;n_{21}=-\frac{a_{210}}{\Delta },\;\;\;n_{22}=\frac{a_{110}}{\Delta },\;\\ n_{23}=\frac{a_{111}a_{210}-a_{211}a_{110}}{\Delta },\;\;\;n_{24}=\frac{a_{121}a_{210}-a_{221}a_{110}}{\Delta },\;\;n_{25}=\frac{a_{150}a_{210}-a_{250}a_{110}}{\Delta },\;\\ n_{28}=\frac{a_{180}a_{210}-a_{280}a_{110}}{\Delta },\;\;\;n_{31}=\frac{1}{a_{660}},\;\;\;n_{32}=-\frac{2a_{661}}{a_{660}},\;\;n_{35}=\frac{a_{350}}{a_{660}},\;\;n_{38}=\frac{a_{380}}{a_{660}},\;\;\;\\ a_{11k_{1}}=\int_{-h/2}^{h/2}Q_{\bar{z}}^{11}z^{k_{1}}dz,\;\;\;a_{12k_{1}}=\int_{-h/2}^{h/2}Q_{\bar{z}}^{12}z^{k_{1}}dz,\;\;\;a_{21k_{1}}=\int_{-h/2}^{h/2}Q_{\bar{z}}^{21}z^{k_{1}}dz,\\ a_{22k_{1}}=\int_{-h/2}^{h/2}Q_{\bar{z}}^{22}z^{k_{1}}dz,\;\;\;a_{66k_{1}}=\int_{-h/2}^{h/2}Q_{\bar{z}}^{66}z^{k_{1}}dz,\;\;\;k_{1}=0,\;1,\;2,\;\;\;a_{15k_{2}}=\int_{-h/2}^{h/2}J_{z}^{1}Q_{\bar{z}}^{11}z^{k_{2}}dz,\;\;\\ a_{18k_{2}}=\int_{-h/2}^{h/2}J_{z}^{2}Q_{\bar{z}}^{12}z^{k_{2}}dz,\;\;a_{25k_{2}}=\int_{-h/2}^{h/2}J_{z}^{1}Q_{\bar{z}}^{21}z^{k_{2}}dz,\;\;a_{28k_{2}}=\int_{-h/2}^{h/2}J_{z}^{2}Q_{\bar{z}}^{22}z^{k_{2}}dz,\;\\ a_{35k_{2}}=\int_{-h/2}^{h/2}J_{z}^{1}Q_{\bar{z}}^{66}z^{k_{2}}dz,\;\;a_{38k_{2}}=\int_{-h/2}^{h/2}J_{z}^{2}Q_{\bar{z}}^{66}z^{k_{2}}dz,\;\;\;k_{2}=0,\;1,\;\;\;\Delta =a_{110}a_{220}-a_{120}a_{210}. \end{array}$$

Here $L_{kp}(i=1,2,3,p=1,2,\ldots ,5)$ are defined as:

(A3) $$\begin{array}{l} L_{11}(\phi )=h\left[(m_{11}-m_{31})\frac{\partial^{4}}{\partial x^{2}\partial y^{2}}+m_{12}\frac{\partial^{4}}{\partial x^{4}}\right],\;\;L_{12}(w)=\rho_{1}\frac{\partial^{4}}{\partial x^{2}\partial t^{2}}+m_{13}\frac{\partial^{4}}{\partial x^{4}}-\left(m_{14}+m_{32}\right)\frac{\partial^{4}}{\partial x^{2}\partial y^{2}},\\ L_{13}(\psi_{1})=m_{15}\frac{\partial^{3}}{\partial x^{3}}+m_{35}\frac{\partial^{3}}{\partial x\partial y^{2}}-J_{3}\frac{\partial }{\partial x}-\rho_{2}\frac{\partial^{3}}{\partial x\partial t^{2}},\;\;L_{14}(\psi_{2})=m_{18}\frac{\partial^{3}}{\partial x^{2}\partial y}+m_{38}\frac{\partial^{3}}{\partial x^{2}\partial y},\\ L_{21}(\phi )=hm_{21}\frac{\partial^{4}}{\partial y^{4}}+h(m_{22}-m_{31})\frac{\partial^{4}}{\partial x^{2}\partial y^{2}},\\ L_{22}(w)=-\left(m_{32}+m_{23}\right)\frac{\partial^{4}}{\partial x^{2}\partial y^{2}}-m_{24}\frac{\partial^{4}}{\partial y^{4}}+\rho_{1}\frac{\partial^{4}}{\partial x^{2}\partial t^{2}},\\ L_{23}(\psi_{1})=m_{35}\frac{\partial^{3}}{\partial x\partial y^{2}}+m_{25}\frac{\partial^{3}}{\partial x\partial y^{2}},\;\;L_{24}(\psi_{2})=m_{38}\frac{\partial^{3}}{\partial x^{2}\partial y}+m_{28}\frac{\partial^{3}}{\partial y^{3}}-J_{4}\frac{\partial }{\partial y}-\rho_{3}\frac{\partial^{3}}{\partial y\partial t^{2}},\\ L_{31}(\phi )=h\left(\frac{1}{s_{2}}\frac{\partial^{2}}{\partial x^{2}}+\frac{1}{s_{1}}\frac{\partial^{2}}{\partial y^{2}}\right),\;\;L_{32}(w)=-\left(V_{*}^{cnt}\rho_{cnt}+V_{m}\rho_{m}\right)h\frac{\partial^{2}}{\partial t^{2}},\;\;\\ L_{33}(\psi_{1})=J_{3}\frac{\partial }{\partial x},\;\;L_{34}(\psi_{2})=J_{4}\frac{\partial }{\partial y},\;\;L_{35}(\phi ,w)=h\left(\frac{\partial^{2}}{\partial y^{2}}\frac{\partial^{2}}{\partial x^{2}}-2\frac{\partial^{2}}{\partial x\partial y}\frac{\partial^{2}}{\partial x\partial y}+\frac{\partial^{2}}{\partial x^{2}}\frac{\partial^{2}}{\partial y^{2}}\right). \end{array}$$

Here $c_{kp}(k=1,2,3,p=1,2,\ldots ,5)$ are described as

(A4) $$\begin{array}{l} c_{11}=i_{1}{}^{2}\left\{\theta_{03}h\delta_{11}\left[(m_{11}-m_{31})j_{1}{}^{2}+m_{12}i_{1}{}^{2}\right]-m_{13}i_{1}{}^{2}-\left(m_{14}+m_{32}\right)j_{1}{}^{2}\right\},\\ c_{11}^{nl}=-\frac{64\theta_{01}hm_{12}}{3ab}\frac{i_{1}{}^{3}}{j_{1}}\left[1-{\left(-1\right)}^{i}-{\left(-1\right)}^{j}+{\left(-1\right)}^{i+j}\right],\\ c_{11}^{t}=-\rho_{1}i_{1}{}^{2},\;c_{12}=i_{1}\left(m_{15}i_{1}{}^{2}+m_{35}j_{1}{}^{2}+J_{3}\right),c_{12}^{t}=\rho_{2}i_{1},c_{13}=(m_{18}+m_{38})i_{1}{}^{2}j_{1},\;\\ c_{21}=j_{1}{}^{2}\left\{\delta_{11}h\theta_{03}\left[m_{21}j_{1}{}^{2}+(m_{22}-m_{31})i_{1}{}^{2}\right]-\left(m_{32}+m_{23}\right)i_{1}{}^{2}-m_{24}j_{1}{}^{2}\right\},\\ c_{21}^{nl}=-\frac{64\theta_{02}m_{21}h}{3ab}\frac{j_{1}{}^{3}}{i_{1}}\left[1-{\left(-1\right)}^{i}-{\left(-1\right)}^{j}+{\left(-1\right)}^{i+j}\right],\;\;\;c_{21}^{t}=-\rho_{1}j_{1}{}^{2},\\ c_{22}=(m_{25}+m_{35})i_{1}j_{1}{}^{2},c_{23}=j_{1}\left(m_{28}j_{1}{}^{2}+m_{38}i_{1}{}^{2}+J_{4}\right),\;\;c_{23}^{t}=\rho_{3}j_{1},\\ c_{31}=\theta_{03}h\delta_{11}\left(\frac{i_{1}{}^{2}}{s_{2}}+\frac{j_{1}{}^{2}}{s_{1}}\right),\;\;c_{32}=2i_{1}{}^{2}j_{1}{}^{2}h\left(\theta_{01}+\theta_{02}\right),\;c_{33}=J_{3}i_{1},\;\;c_{34}=J_{4}j_{1}\\ c_{31}^{nl}=-\frac{8h}{3ab}\left[2\left(\frac{\theta_{01}}{s_{2}}\frac{i_{1}}{j_{1}}+\frac{\theta_{02}}{s_{1}}\frac{j_{1}}{i_{1}}\right)+i_{1}j_{1}\delta_{11}\theta_{03}\right]\left[1-{\left(-1\right)}^{i}-{\left(-1\right)}^{j}+{\left(-1\right)}^{i+j}\right], \end{array}$$

where

(A5) $$\theta_{01}=\frac{\delta_{14}}{32i_{1}^{4}\delta_{3}},\;\;\;\theta_{02}=\frac{\delta_{14}}{32j_{1}^{4}\delta_{1}},\;\;\;\;\theta_{03}=\frac{1}{\delta_{1}j_{1}^{4}+\delta_{2}i_{1}^{2}j_{1}^{2}+\delta_{3}i_{1}^{4}}$$
